# Psychotic Experiences and Working Memory: A Population-Based Study Using Signal-Detection Analysis

**DOI:** 10.1371/journal.pone.0153148

**Published:** 2016-04-27

**Authors:** Rodolfo Rossi, Stanley Zammit, Katherine S. Button, Marcus R. Munafò, Glyn Lewis, Anthony S. David

**Affiliations:** 1 Section of Psychiatry, University School of Medicine Federico II, Naples, Italy; 2 Section of Cognitive Neuropsychiatry, Institute of Psychiatry, Psychology and Neuroscience, King’s College, London, United Kingdom; 3 School of Social and Community Medicine, University of Bristol, Bristol, United Kingdom; 4 Institute of Psychological Medicine and Clinical Neurosciences, Cardiff University, Cardiff, United Kingdom; 5 Department of Psychology, University of Bath, Bath, United Kingdom; 6 UK Centre for Tobacco and Alcohol Studies, School of Experimental Psychology, University of Bristol, Bristol, United Kingdom; 7 MRC Integrative Epidemiology Unit (IEU) at the University of Bristol, Bristol, United Kingdom; 8 Division of Psychiatry, University College London, London, United Kingdom; University of Chicago, UNITED STATES

## Abstract

Psychotic Experiences (PEs) during adolescence index increased risk for psychotic disorders and schizophrenia in adult life. Working memory (WM) deficits are a core feature of these disorders. Our objective was to examine the relationship between PEs and WM in a general population sample of young people in a case control study. 4744 individuals of age 17–18 from Bristol and surrounding areas (UK) were analyzed in a cross-sectional study nested within the Avon Longitudinal Study of Parents and Children (ALSPAC) birth cohort study. The dependent variable was PEs, assessed using the semi-structured Psychosis-Like Symptom Interview (PLIKSi). The independent variable was performance on a computerized numerical n-back working memory task. Signal-Detection Theory indices, including standardized hits rate, false alarms rate, discriminability index (*d’*) and response bias (*c*) from 2-Back and 3-Back tasks were calculated. 3576 and 3527 individuals had complete data for 2-Back and 3-Back respectively. Suspected/definite PEs prevalence was 7.9% (N = 374). Strongest evidence of association was seen between PEs and false alarms on the 2-Back, (odds ratio (OR) = 1.17 [95% confidence intervals (CI) 1.01, 1.35]) and 3-back (OR = 1.35 [1.18, 1.54]) and with *c* (OR = 1.59 [1.09, 2.34]), and lower *d’* (OR = 0.76 [0.65, 0.89]), on the 3-Back. Adjustment for several potential confounders, including general IQ, drug exposure and different psycho-social factors, and subsequent multiple imputation of missing data did not materially alter the results. WM is impaired in young people with PEs in the general population. False alarms, rather than poor accuracy, are more closely related to PEs. Such impairment is consistent with different neuropsychological models of psychosis focusing on signal-to-noise discrimination, probabilistic reasoning and impaired reality monitoring as a basis of psychotic symptoms.

## Introduction

### Background

Psychotic Experiences (PEs) such as delusions and hallucinations are relatively common and not necessarily associated with a clinical condition in the general population. PEs estimated lifetime prevalence is about 2 to 3 times [1, 2] that of psychotic disorder (approximately 2.9%[3]). Estimates vary according to method of assessment, from 5% when assessed using semi-structured interview[4, 5] to 60% when using self-report measures[6]. PEs reach a prevalence peak during early adolescence subsiding to a plateau in early adulthood[1, 7–9], although the exact rate of persistence of PE is not known. PEs index increased risk for full-blown psychotic disorder and schizophrenia[10] although estimates of rate of conversion suggest that most individuals with PEs do not convert to a psychotic disorder[1]. PEs share some of the same underlying risk factors as psychotic disorders, including drug abuse, environmental stressors, neurodevelopmental factors and family history of psychiatric conditions[11, 12].

One well established association with psychotic disorders, including schizophrenia, is cognitive impairment, in particular of working memory. Working memory (WM) is a core cognitive function commonly found to be impaired across the schizophrenia spectrum and it represents a candidate endophenotype for schizophrenia [13–15]. In fact, WM deficits have been confirmed both before and after the first episode of the disorder[16–18]. Individuals with schizophrenia seem to follow specific deterioration trajectories in WM throughout their lives[17, 19, 20]. Furthermore, minor WM impairment is evident in first-degree relatives of people with schizophrenia and other individuals at familial-risk[21–23], suggesting a genetic contribution[24, 25]. Reduced WM capacity has also been found in those help-seeking individuals deemed to be at Ultra-High Risk (UHR)[26] or to have an At-Risk Mental State (ARMS) for psychosis, especially in those who convert to psychotic disorder[27–30]. Finally, a large body of work reports on the association of WM deficits with schizotypy with mixed results, possibly due to the heterogeneity of the instruments used [31].

The putative neuropsychological mechanisms by which WM deficits contribute to psychosis are debated. WM has been related to specific reasoning and neurocognitive anomalies, including source-monitoring or source-memory disturbances[32–34] and reasoning biases like jumping-to-conclusions (JTC)[35–37]. As a whole, psychotic phenomena are associated with a high rate of false memories and/or a failure to reject them[38, 39] culminating in impaired reality monitoring [40].

A few studies have addressed the relationship between WM and PEs in the general population. For example, Korponay and colleagues[41] in the US found unexpectedly higher WM scores, assessed using the MATRICS Consensus Cognitive Battery (MCCB) in *adult* individuals experiencing positive sub-clinical psychotic symptoms defined using the Community Assessment of Psychic Experiences (CAPE) self-report questionnaire. However, concerns may be raised about the high mean age of this sample and its suitability to address the relationship between neurocognitive functioning and PE as a risk factor for psychosis, since such individuals may be resistant to transition to a psychotic disorder. An association between WM and PEs, again assessed using the CAPE, was also found in a large population study in Germany [42]. In this study WM was assessed using two tasks that do not permit signal detection analysis. A statistically robust correlation between WM capacity and PEs was detected, although its effect size was deemed small. Both studies addressed PEs using a self-report instrument, which may have overestimated the prevalence of PEs. In Ireland, a small interview-based study of PEs found no significant differences in WM capacity between adolescent cases and controls measured using the digit span task[43]. The same group also reported separately [44] a significant correlation between individuals with PEs and spatial WM. In the same cohort, a subsample of individuals with a prodromal syndrome showed a similar deficit in spatial WM tasks [45].

### Objectives

Here we examine WM functioning using a version of the N-back task on the largest sample to date of around 4000 adolescents[5, 25, 46] within a signal-detection theory (SDT) framework. These participants came from a population-based birth cohort and were assessed using a psychologist administered semi-structured interview for PEs[5]. SDT describes the probabilistic processes of decision-making under conditions of uncertainty [47], and it is particularly useful for our purposes as it allows us to characterize features of WM functioning beyond simple WM capacity. Indeed, early uses of SDT revealed that hallucinatory proneness as well as hallucinatory experiences, are highly correlated with false recognitions, detected as ‘false alarms’ and a liberal response bias in an on-line SDT task (see below)[48].

We sought to test the overall hypothesis that there is continuity between PEs and psychotic disorders; hence WM anomalies should show an association with PEs despite the absence of clinical disorder. Our sample allowed us to test this association without the confounding effect of treatment, illness or help-seeking behaviour. We reasoned that if confirmed, this would enable us to better characterize which specific components in a WM task are related to the PEs-psychosis continuum.

## Material and Methods

### Sample

We examined data collected from the Avon Longitudinal Study of Parents and Children (ALSPAC) cohort[49]. ALSPAC is a population pre-birth cohort study of all the pregnancies in the Avon county, UK, with deliveries between 1 April 1991 and 31 December 1992[49, 50]. In total, 14,893 mothers enrolled, representing an estimated 85–90% of the eligible population. We selected cases from the “Focus on Teen 4” time-point, scheduled at age 17 (N = 5216) (for further details on data collected, see http://www.bristol.ac.uk/alspac/researchers/resources-available/). Of these, those who had completed either the interview for PEs or the N-Back task (see later) were selected. Participants provided written consent for data collection and analysis. Ethical approval was obtained from the ALSPAC Law and Ethics Committee and the local research ethics committees (http://www.bristol.ac.uk/alspac/researchers/data-access/ethics/). The research adheres to the tenets of the Declaration of Helsinki.

### Measures

#### Psychotic Experiences

The presence of PEs was assessed using the semi-structured Psychosis-Like Symptom Interview (PLIKSi)[8]. The PLIKSi comprises an introductory set of questions to accustom the participant to probes for unusual experiences, followed by 11 “core” questions, based on, and rated according to guidelines for the WHO Schedules for Clinical Assessment in Neuropsychiatry version 2.0 (SCAN 2.0[51]).

PEs included hallucinations (visual and auditory), delusions (spied on, persecution, thoughts read, reference, control and grandiosity) and experiences of thought interference (broadcasting, insertion and withdrawal). Each PE was rated by the interviewer as “Absent”, “Suspected” or “Definitely Present”. Unclear or ambiguous responses were rated down. The PE was rated as “Definitely Present” only if a clear example was provided. In this study individuals were classed as having a PE if rated as having any “Suspected” or “Definite” PE, which was not attributed to sleep or fever. PLIKSi at age 12 showed good psychometric properties [5, 8].

#### Working Memory

WM was assessed using an N-Back task (for details see[25]). 3986 individuals had available N-Back data. Of these, 391 participants for the 2-Back and 341 for the 3-Back were excluded due to non-responsiveness to the task. Subjects were presented with a series of 0–9 numbers on a computer screen, each presented for 500ms with an inter-stimulus interval of 3000ms. Subjects were instructed to press button “1” if the number was the same as the number of N numbers before, “2” if it was different. Instructions provided to participants were neutral with respect to speed or accuracy. Two different blocks (2-Back and 3-Back) were administered, each of 48 trials. Four different parameters were automatically generated by the computer:

*a*. target identification accuracy (Hits), being the percentage of trials correctly identified as targets, *b*. non-target identification accuracy, being the percentage of trials correctly identified as non-targets, *c*. target identification median reaction time (Hits RT), *d*. non-target identification median reaction time (nonT RT).

### Signal Detection Theory (SDT)

In a SDT-based task, subjects are required to detect the presence (or absence) of a target stimulus under conditions of relative uncertainty [47, 52]. According to SDT, on a given trial in which a target stimulus or noise can be alternatively presented, subjects respond according to the value that an inner unobserved decision variable achieves during each trial. If the decision variable exceeds a certain threshold, which is called *criterion*, the subject detects the target (i.e. responds “*yes*”); otherwise, the subject does not detect the target (i.e. responds “*no”*) [53]. Among the various performance indices derivable from SDT, we chose to analyse hits and false alarms (FA) separately, the discriminability index *d’* as a global measure of performance, and response bias *c*, defined as the amount of certainty needed to make a decision on the response [54], or, given the same amount of uncertainty, the bias towards detecting (or rejecting) a stimulus as a target (a low value of *c* means that less information is needed to detect the target). Response bias *c* can be conceptualized as an index of a liberal (c<0) vs cautious (c>0) or neutral (c = 0) decision-making strategy [55].

#### SDT measures

From the four raw scores, False Alarms (FA) were calculated as:

FA = 1 - (non target identification accuracy)

Standardized scores for Hits (z-Hits), FA (z-FA), Hits RT (z-Hits RT) and nonT RT (z-nonT RT) were calculated and included in the analysis.

Discriminability index *d’* was calculated using the Stata syntax (adapted from[53]):

d’ = invnorm(H)– invnorm(FA)

Response bias *c* was calculated using the Stata syntax (adapted from[53]):

c = - (invonrm(Hits))+ (invonrm(FA))/2

### Confounders

A number of potential socio-demographic confounders were selected *a priori* from the whole database, according to previously published papers from the same cohort. These were: ICD-10 diagnosis of depression, assessed using a computerized version of the Clinical Interview Schedule (CIS-R)[56, 57], bullying profile (bully/victim/bully and victim), standardized Total IQ measured at age 8 using an abbreviated form of the Wechsler Intelligence Scale for Children—III (WISC)(UK version)[58], Family Adversity Index (FAI) [59], cannabis abuse, assessed using the Cannabis Abuse Screening Test (CAST) [60, 61] referring to the last 6 months before the interview (coded as positive if CAST score was ≥ 1, absent if subject had never tried cannabis before or if CAST score = 0), use of any hard drug during previous 3 months; Alcohol Use Disorder Identification Test (AUDIT) scores; and gender.

### Statistical Analysis

All statistical analyses were conducted using Stata v. 13 (StataCorp, College Station, Texas). Frequencies for categorical variables, means and 95% Confidence Intervals (95% CIs) for continuous variable were calculated. A Wilcoxon signed-rank test for repeated measures was used to compare the performance on the 2-Back vs. 3-Back. In a first wave of analyses, univariable logistic regression was used to calculate unadjusted Odds Ratios (OR) and 95% CIs for PE outcomes in relation to the different N-Back parameters.

In a second wave of analyses, a two-block nested multiple logistic regression was carried out in order to separate the effect of confounding from missing data. In the first block, unadjusted ORs were calculated for individual N-Back parameters on a sample restricted to those having complete data for the confounders. In the second block, the aforementioned confounders were introduced in the model on the same sample.

Missing data patterns were examined. Missing data were imputed using Multiple Imputation by Chained Equation (MICE) methods using the–ice- command in Stata[62]. Imputation was performed on the subsample initially selected for analysis (N = 4744).

## Results

### Sample

A total of N = 4744 (90.95%) individuals was selected for analysis, having attended the session and having complete data for at least one of PLIKSi or N-Back. Of these, 56.5% [55.1, 57.9] were female and 95.7% [95.1, 96.3] were of white ethnicity. The mean age of the sample was 17.78 years [17.77, 17.79], mean IQ was 107.3 [106.8, 107.8]. Details of other variables in the sample are reported in Table 1.

**Table 1:** Participant Characteristics.

	Overall (Mean [95%CI] or Count (%))	Non-PEs (Mean [95%CI] or Count (%))	PEs (Mean [95%CI] or Count (%))	Effect Size (Cohen’s d or Cramer’s V)
***Sex (Female)***	2681 (56.5%)	2425 (55.8%)	240 (64.2%)	-0.04
***Age (years)***	17.77 [17.76, 17.78]	17.77 [17.76, 17.78]	17.81 [17.77, 17.85]	-0.11
***Total IQ***	107.3 [106.80, 107.81]	107.54 [107.01, 108.07]	104.17 [102.30, 106. 04]	0.20
***Highest Parental Social Class***				
I+II	2646 (63.93%)	2,469 (64.89%)	166 (53.21%)	0.07
III non manual	964 (23.3%)	870 (22.86%)	86 (27.56%)	
III manual	367 (8.90%)	326 (8.57%)	38 (12.18%)	
IV or V	162 (3.91%)	140 (3.68%)	22 (7.05%)	
***ICD-10 Depression***	335 (7.71%)	257 (6.41%)	78 (23.08%)	0.16
***Family Adversity Index***	1.01 [0.97, 1.05]	0.97 [0.93, 1.01]	1.50 [1.31, 1.68]	-0.37
***Cannabis (yes)***	165 (4.75%)	123 (3.81%)	42 (16.34%)	0.15
***A*.*U*.*D*.*I*.*T (yes)***	1571 (42.36%)	1433 (41.9%)	137 (47.9%)	0.03
***Hard Drug Use (yes)***	206 (5.70%)	165 (4.8%)	41 (17.15%)	0.13
***Bullying***				
Bullied	616 (15.75%)	551 (15.21%)	65 (22.57%)	0.11
Bully	155 (3.96%)	143 (3.95%)	12 (4.17%)	
Bullied/Bully	457 (11.68%)	395 (10.9%)	62 (21.53%)	

### PLIKSi

A total of 4718 cases had complete PLIKSi data. Of these, 7.9% [7.19, 8.73] (N = 374) had reported at least one suspected or definite PE. Symptom-positive participants were 64.2% [59.3, 69.0] female; 93.5% [90.8, 96.2] were of white ethnicity. Their mean IQ was 104.2 [102.4, 106.0], slightly lower than symptom-free individuals (107.6 [107.0, 108.1]). Detailed prevalence for each of the single PLIKSi item is reported in the Table 2.

**Table 2:** Details of PLIKSi Items and their prevalence (count and proportion) in our sample.

PLIKS Item	None	Suspect	Definite	Any (Suspect + Definitive)
**1. *Auditory Hallucinations***	4465 (94.66)	107 (2.27)	145 (3.07)	252 (5.34)
**2. *Visual Hallucinations***	4516 (95.74)	88 (1.87)	112 (2.37)	200 (4.24)
**3. *Visual Illusions***	4654 (98.66)	34 (0.72)	29 (0.61)	63 (1.33)
**4. *Delusions Of Being Spied On***	4635 (98.24)	55 (1.17)	26 (0.55)	81 (1.72)
**5. *Delusions Of Persecution***	4679 (99.17)	22 (0.47)	12 (0.25)	34 (0.72)
**6. *Delusions Of Thoughts Being Read***	4700 (99.62)	12 (0.25)	6 (0.13)	18 (0.38)
**7. *Delusions Of Reference***	4685 (99.32)	23 (0.49)	8 (0.17)	31 (0.66)
**8. *Delusions Of Control***	4708 (99.79)	6 (0.13)	4 (0.08)	10 (0.21)
**9. *Delusions Of Grandiose Ability***	4696 (99.53)	13 (0.28)	5 (0.11)	18 (0.39)
**10. *Thought Broadcasting***	4677 (99.24)	28 (0.59)	8 (0.17)	36 (0.76)
**11. *Thought Insertion***	4685 (99.41)	15 (0.32)	13 (0.28)	28 (0.6)
**12. *Thought Withdrawal***	4708 (99.89)	4 (0.08)	1 (0.02)	5 (0.1)

### Working Memory

2-Back and 3-Back scores were available for 3595 and 3551 individuals, respectively. All performance parameters differed between 2-back and 3-back procedures, with individuals having lower Hits, and *c*, and higher FA on the 3-back task. A detailed description of the individual N-Back parameters is reported in Table 3.

**Table 3:** Mean, 95% CI and Range of performance on 2-Back and 3-Back.

	Mean	Mean 95% CI	Range	Mean	Mean 95% CI	Range
	*2-Back (N = 3595)*			*3-Back (N = 3551)*		
Hits	0.71	[0.71, 0.72]	0.13, 0.94	0.56	[0.55, 0.57]	0.13, 0.94
FA	0.21	[0.20, 0.21]	0.01, 0.97	0.22	[0.21, 0.22]	0.01, 0.97
Hits RT	702.84	[695.11, 710.57]	139.50, 2002.50	737.12	[727.20, 747.04]	74.5, 2410
nonT RT	671.91	[664.92, 678.89]	6.50, 1858.50	696.94	[688.27, 705.61]	32, 2278
d’	1.73	[1.69, 1.77]	-3.01, 3.79	1.12	[1.08, 1.15]	-2.77, 3.79
c^a^	0.19	[0.17, 0.21]	-1.70, 1.50	0.37	[0.35, 0.38]	-1.53, 1.70

^a^ negative values of c signify liberal bias, whereas positive values signify conservative bias.

### Univariable Logistic Regression

In the crude model, of the 2-Back parameters investigated, higher z-FA rate was associated with PEs (OR = 1.17 [1.01,1.35]). On the 3-Back task, higher z-FA rate (OR = 1.35 [1.18,1.54]) and *c* (OR = 1.59 [1.09,2.34]), and lower *d’* (OR = 0.76 [0.65,0.89]) were significantly associated with PEs (Table 4).

**Table 4:** Unadjusted and adjusted Odds Ratios, p value, and 95% CI of individual N-Back parameters for participants with PEs versus those without Pes.

N-Back Parameters	OR	p	95%CI	OR	p	95%CI	OR	p	95%CI
	*2-Back*, *Unadjusted (N = 3576)*			*2-Back block 1 (N = 1970)*			*2-Back*, *adjusted*^a^ *(N = 1970)*		
z-Hits	0.94	0.445	[0.80,1.10]	0.93	0.549	[0.74, 1.17]	0.96	0.738	[0.76,1.22]
z-False Alarms	**1.17**	**0.032**	**[1.01,1.35]**	1.05	0.687	[0.84, 1.31]	0.95	0.706	[0.74,1.22]
*d’*	0.90	0.084	[0.80,1.01]	0.96	0.694	[0.81, 1.15]	1.03	0.753	[0.85,1.25]
c^b^	0.79	0.173	[0.56,1.11]	1.08	0.786	[0.63, 1.82]	1.25	0.423	[0.73,2.14]
z-Hits RT	1.01	0.871	[0.86,1.19]	1.01	0.939	[0.80, 1.28]	1.00	0.984	[0.79,1.26]
z-nonT RT	1.03	0.693	[0.88,1.21]	1.08	0.515	[0.86, 1.35]	1.10	0.431	[0.87,1.38]
	*3-Back*, *Unadjusted (N = 3527)*			*3-Back block 1 (N = 1947)*			*3-Back*, *adjusted*^a^ *(N = 1947)*		
z-Hits	0.91	0.227	[0.77,1.06]	0.90	0.350	[0.71, 1.13]	0.92	0.494	[0.73,1.17]
z-False Alarms	**1.35**	**0.001**	**[1.18,1.54]**	**1.26**	**0.023**	**[1.03, 1.54]**	1.19	0.106	[0.96,1.48]
*d’*	**0.76**	**0.001**	**[0.65,0.89]**	**0.80**	**0.049**	**[0.64, 1.00]**	0.84	0.150	[0.67,1.06]
c^‡^	**0.63**	**0.017**	**[0.43,0.92]**	0.75	0.322	[0.43, 1.32]	0.84	0.528	[0.48,1.46]
z-Hits RT	0.87	0.127	[0.74,1.04]	0.85	0.197	[0.66, 1.09]	0.89	0.391	[0.69,1.16]
z-nonT RT	0.94	0.466	[0.80,1.11]	0.90	0.381	[0.71, 1.14]	0.93	0.580	[0.73,1.19]

^a^ adjusted for: ICD-10 Diagnosis of depression, Bullying profile, Total IQ at age 8, Family Adversity Index, cannabis abuse, Hard Drugs use, Gender

^b^ Negative values of c signify liberal bias, whereas positive values signify conservative bias.

### Nested Regression

In the nested logistic regression, the sample size dropped from N = 3576 to N = 1970 and from N = 3527 to N = 1947 for the 2-Back and 3-Back respectively, due to missing data. In the first unadjusted block, only the 3-Back z-FA and *d’* showed an association with PEs, respectively: OR = 1.26 [1.03, 1.54] and 0.8 [0.64, 1.00]. No significant association between N-Back performance or response bias on the two N-Back tasks and PEs was found in the adjusted model (Table 4).

### Multiple imputation

After multiple imputation, our sample size was N = 4744. The results were similar to the univariable unadjusted model, suggesting that attrition may have affected standard errors in the adjusted models. In the 2-Back unadjusted model, z-FA and *d’* were associated with PEs (respectively, OR = 1.17 [1.01, 1.34] and OR = 0.89 [0.79, 0.99]). In the 3-Back unadjusted model z-FA (OR = 1.36 [1.20, 1.55]), *d’* (OR = 0.74 [0.64, 0.86]), and response bias *c* (OR = 0.64 [0.43, 0.93]) showed a significant association with PEs. In the adjusted imputed model, only on the 3-Back, z-FA (OR = 1.25 [1.08, 1.44]) and *d’* (OR = 0.82 [0.69, 0.96]) showed an association with PEs (Table 5).

**Table 5:** Unadjusted and adjusted Odds Ratios, p value, and 95% CI of individual N-Back parameters for participants with PEs—Multiple Imputation.

N-Back Parameters	OR	p	95%CI	OR	p	95%CI
	*2-Back unadjusted MICE (N = 4744)*			*2-Back adjusted*^a^ *MICE (N = 4744)*		
z-Hits	0.92	0.292	[0.78, 1.08]	1.00	0.965	[0.84, 1.19]
z-False Alarms	**1.17**	**0.034**	**[1.01, 1.34]**	1.03	0.721	[0.88, 1.21]
*d’*	**0.89**	**0.040**	**[0.79, 0.99]**	0.99	0.841	[0.87, 1.13]
c^b^	0.82	0.317	[0.56, 1.22]	0.96	0.852	[0.66, 1.42]
z-Hits RT	1.00	0.987	[0.83, 1.20]	1.03	0.755	[0.85, 1.24]
z-nonT RT	1.04	0.633	[0.88, 1.23]	1.08	0.360	[0.91, 1.29]
	*3-Back unadjusted MICE (N = 4744)*			*3-Back adjusted*^a^ *MICE (N = 4744)*		
z-Hits	0.88	0.118	[0.75, 1.03]	0.94	0.465	[0.79, 1.12]
z-False Alarms	**1.36**	**<0.001**	**[1.20, 1.55]**	**1.25**	**0.003**	**[1.08, 1.44]**
*d’*	**0.74**	**<0.001**	**[0.64, 0.86]**	**0.82**	**0.015**	**[0.69, 0.96]**
c^b^	**0.64**	**0.022**	**[0.43, 0.93]**	0.73	0.117	[0.49, 1.09]
z-Hits RT	0.85	0.118	[0.69, 1.04]	0.92	0.383	[0.75, 1.12]
z-nonT RT	0.92	0.339	[0.77, 1.10]	1.00	0.982	[0.83, 1.20]

^a^ adjusted for: ICD-9 Diagnosis of depression, Bullying profile, Total IQ at age 8, Family Adversity Index, cannabis abuse, Hard Drugs use, Gender

^b^ negative values of c signify liberal bias, whereas positive values signify conservative bias.

## Discussion

This study clarifies evidence of impaired WM performance in individuals with PEs in a general population birth cohort. Our findings replicate and extend previous studies[41, 63]. Compared to those studies, we report on a larger sample size, and we used an interview-based assessment method for PEs. Furthermore, we used SDT to obtain a fine-grained profiling of WM unlike previous studies that, for example, used the MATRICS Consensus Cognitive Battery[64] to assess global cognitive functioning[41].

Our data show that poor performance on the 3-Back paradigm was more strongly associated with PEs than was the 2-Back, suggesting that the WM deficit emerges in a PE-positive population only at higher WM loads. This could be related to the narrower WM span demonstrated in psychotic disorders and schizophrenia[65–67], although the same association is hitherto less established with PE [41–45]. In our sample, the association of impaired WM with PEs was only partially confounded by general intelligence, as shown in the multiple imputation analysis. Moreover, the loss of association between WM and PEs after adjusting for potential confounders may be due, at least in part, to the drop in sample size, as highlighted in the nested block 1 model. Hence, we judge the overall confounding effect to be relatively small, although not null.

In our study, false alarms showed the strongest association with PEs, suggesting that the performance on this task is being dragged down by poor accuracy in rejecting non-targets rather than poor accuracy in detecting targets. This result, together with a liberal response bias, is consistent with findings of an association between increased false recognitions and psychotic phenomena. For example, a similar SDT profile, with diminished discriminability and a liberal response bias, has been found to be associated with positive schizotypy in two studies [68, 69]. In a recent study, individuals with schizophrenia displayed a similar SDT pattern in two similar tasks with higher rates of false alarms and diminished d’ compared to controls [70]. Moreover, auditory hallucinations were found to be associated with visual memory errors [71], false recognition of auditory signals [48, 72] and words [73]. Taken together, these findings suggest that liberal response bias and diminished discriminability are general non-mnemonic characteristics underlying impaired reality-testing and positive psychotic phenomena.

A liberal response bias (lower value of *c*) could be viewed as an impulsive style of responding [74]. This would imply a correlation between reaction time and response bias. In our study, neither speed nor accuracy was favoured in the task instructions. However, we carried out a post-hoc correlation analysis (Table 6) and found a negative correlation between reaction time and false alarms (ie., faster response, more false alarms) and a positive correlation with hits, for the 3–back task. While this might suggest impulsivity, there was no correlation between *c* and reaction time. Experimental manipulation of speed and accuracy may shed light on this issue and relevance to the formation of PEs [75] in future studies.

**Table 6:** N-Back indices Pearson Correlations.

	z-Hits	z-False Alarms	z-Hits RT	z-nonT RT	c
**2-Back (N = 3594)**					
z-False Alarms	-0.313***				
z-Hits RT	0.212***	-0.171***			
z-nonT RT	0.244***	-0.200***	0.775***		
c	-0.433***	-0.691***	0.001	-0.014	
d’	0.782***	-0.811***	0.221***	0.251***	0.208***
**3-Back (N = 3550)**					
z-False Alarms	-0.201***				
z-Hits RT	0.328***	-0.283***			
z-nonT RT	0.349***	-0.292***	0.812***		
c	-0.626***	-0.610***	-0.038*	-0.053**	
d’	0.788***	-0.736***	0.378***	0.400***	-0.025

* p<0.05,

** p<0.01,

*** p<0.001

### Theoretical implications

Our SDT-based dissection of WM impairment is consistent with other neuropsychological models of psychosis. The relevance of poor accuracy in rejecting non-targets in PEs, rather than inhibiting the response, is confirmed by studies on auditory hallucinations. According to the SDT-based framework, hallucinations can be conceptualized as false alarm-equivalents, in the sense that the absence of a stimulus elicits a response as if that stimulus was actually present [76]. In fact, it has indeed been found that auditory hallucinations are related to poor discriminability and liberal response bias [77, 78]. The response bias, in turn, could be influenced by aberrantly hypervigilant attentional systems, affecting the rate of false alarms [79, 80]. The importance of attentional systems has been addressed by Cohen and colleagues [81–83] using the Continuous Performance Test (CPT). Our results are in line with theirs, in terms of SDT. However, attention based SDT-models focus on the processing of present stimuli or on lower loads of WM. We are assuming that both mnemonic and online representations of stimuli are governed by similar processes, hence similar response biases to those shown on WM tasks but based on ongoing perceptual inputs could lead to judgment errors and possibly PEs.

Our finding of a liberal response bias and a high rate of false alarms associated with PEs is consistent with neurocognitive models of psychosis involving bias in data gathering, including the Jumping-To-Conclusions (JTC) model of delusions[84–86]. This reasoning bias has also been revealed in individuals at risk for psychosis[85, 87] and in delusion-prone individuals[88, 89], suggesting that data-gathering may be impaired before the onset of full-blown psychosis. Moreover, an association between JTC and WM has been reported[35–37, 85] although this has not been addressed in terms of SDT.

A unifying model that puts together data gathering, sensory processing and SDT, that could eventually explain the role of WM deficits in producing PEs is derived from computational neurosciences[90–92]. In computational terms, high “cognitive noise” is associated with reduced *precision* of high-level representations, and with increased relevance and strength of new sensory evidence, eventually leading to psychotic symptoms[91]. Computational models of WM have suggested that the net effect of the putative dopaminergic perturbation occurring in the dorsolateral prefrontal cortex (dlPFC) results in increased “cognitive noise” and impaired gating[93, 94], causing defective stimulus discriminability[95].

Moreover, dopaminergic perturbations in dlPFC produce a persistent state of “WM activation” that, together with impaired gating functions, results in higher error-rates[93]. These mechanisms foster false identification of the present sensory (bottom-up) stimulus as matching the preceding (top-down) one, resulting in a false alarm response.

Finally, the tendency of over-responding in the face of uncertainty, expressed by a liberal response bias, could be explained by the Bayesian concept of precision[90] and metacognitive strength of “prior belief”. In this case, the subject would attribute excessive precision, or is too confident in his mental representations, regardless of the degree of uncertainty associated with them[91, 96]. Such over-confidence may underlie reasoning biases such as JTC[97].

Testing such models would benefit from manipulation of reward/punishment as well as speed/accuracy trade offs[98].

### Strengths and limitations

Our project replicates several previous findings. Although the subject of this report has been extensively addressed before, to date no other work has reported on such sample size. Moreover, no other work in this field has used such a comprehensive SDT approach, detailing some of the psycho-mathematical features of reality processing in subjects with PEs.

One major limitation of our study is the narrow focus on WM, and on N-back. The N-Back task explores, by its nature, only some aspects of the neural mechanism (i.e. encoding and updating) underlying WM as a whole. However, the use of n-back allowed us to dissect some of the non-purely mnemonic aspects involved in performing a WM task, which may have implications for other similar tasks. Indeed, our results are largely consistent with results of Continuous Performance Tasks, of which the n-back is a variation, on the psychosis continuum. Assessing the WM profile in the context of a wider cognitive screening could have helped establish the mutual relationship between different cognitive domains.

A second limitation of ALSPAC is the extent of attrition with under-representation of lower socio-economic groups and ethnic minorities [5] and consequent potential selection bias. However, we used multiple imputation of missing data to help address attrition bias.

The present study has several strengths.

Firstly, as noted, this study is based on a considerably larger sample than any previous study. Secondly, we focused on a non-clinical sample. A large body of work has confirmed WM deficits in clinical populations of ARMS, UHR or FEP [16, 28, 99]. Such clinical samples may be subject to selection bias towards help-seeking and perhaps poor coping strategies. Studying PEs in the general population reduces selection bias based on clinical severity and the effects of medication. Thirdly, PEs were assessed with a semi-structured interview derived from widely-used diagnostic interviews, rather than relying on self-report measures. Fourthly, our study includes a wealth of other demographic and clinical data, allowing us to adjust for potential confounders and better interpret the results.

## Conclusion

In conclusion, this study confirms and extends prior reports of cognitive impairments in individuals with PEs. Our approach using SDT suggests critical connections between different underpinnings of psychosis, including reasoning biases and probabilistic reasoning, and WM functioning.

## References

[1] Linscott RJ, van Os J. An updated and conservative systematic review and meta-analysis of epidemiological evidence on psychotic experiences in children and adults: on the pathway from proneness to persistence to dimensional expression across mental disorders. Psychological medicine. 2013;43(6):1133–49. 10.1017/S0033291712001626 . 22850401

[2] McGrath JJ, Saha S, Al-Hamzawi A, Alonso J, Bromet EJ, Bruffaerts R, et al Psychotic Experiences in the General Population: A Cross-National Analysis Based on 31261 Respondents From 18 Countries. JAMA psychiatry. 2015 10.1001/jamapsychiatry.2015.0575 . 26018466 PMC5120396

[3] Perala J, Suvisaari J, Saarni SI, Kuoppasalmi K, Isometsa E, Pirkola S, et al Lifetime prevalence of psychotic and bipolar I disorders in a general population. Archives of general psychiatry. 2007;64(1):19–28. 10.1001/archpsyc.64.1.19 . 17199051

[4] Polanczyk G, Moffitt TE, Arseneault L, Cannon M, Ambler A, Keefe RS, et al Etiological and clinical features of childhood psychotic symptoms: results from a birth cohort. Archives of general psychiatry. 2010;67(4):328–38. 10.1001/archgenpsychiatry.2010.14 20368509 PMC3776482

[5] Horwood J, Salvi G, Thomas K, Duffy L, Gunnell D, Hollis C, et al IQ and non-clinical psychotic symptoms in 12-year-olds: results from the ALSPAC birth cohort. The British journal of psychiatry: the journal of mental science. 2008;193(3):185–91. 10.1192/bjp.bp.108.051904 18757973 PMC2806573

[6] Laurens KR, Hodgins S, Maughan B, Murray RM, Rutter ML, Taylor EA. Community screening for psychotic-like experiences and other putative antecedents of schizophrenia in children aged 9–12 years. Schizophrenia research. 2007;90(1–3):130–46. 10.1016/j.schres.2006.11.006 . 17207968

[7] Thapar A, Heron J, Jones RB, Owen MJ, Lewis G, Zammit S. Trajectories of change in self-reported psychotic-like experiences in childhood and adolescence. Schizophrenia research. 2012;140(1–3):104–9. 10.1016/j.schres.2012.06.024 . 22789670

[8] Zammit S, Kounali D, Cannon M, David AS, Gunnell D, Heron J, et al Psychotic experiences and psychotic disorders at age 18 in relation to psychotic experiences at age 12 in a longitudinal population-based cohort study. The American journal of psychiatry. 2013;170(7):742–50. 10.1176/appi.ajp.2013.12060768 . 23639948

[9] Mollon J, David AS, Morgan C, Frissa S, Glahn D, Pilecka I, et al Psychotic Experiences and Neuropsychological Functioning in a Population Sample. JAMA psychiatry in press. 10.1001/jamapsychiatry.2015.2551 26720073

[10] Kaymaz N, Drukker M, Lieb R, Wittchen HU, Werbeloff N, Weiser M, et al Do subthreshold psychotic experiences predict clinical outcomes in unselected non-help-seeking population-based samples? A systematic review and meta-analysis, enriched with new results. Psychological medicine. 2012;42(11):2239–53. 10.1017/S0033291711002911 . 22260930

[11] Kounali D, Zammit S, Wiles N, Sullivan S, Cannon M, Stochl J, et al Common versus psychopathology-specific risk factors for psychotic experiences and depression during adolescence. Psychological medicine. 2014;44(12):2557–66. 10.1017/S0033291714000026 25055173 PMC4108252

[12] Stochl J, Khandaker GM, Lewis G, Perez J, Goodyer IM, Zammit S, et al Mood, anxiety and psychotic phenomena measure a common psychopathological factor. Psychological medicine. 2015;45(7):1483–93. 10.1017/S003329171400261X . 25394403

[13] Park S, Gooding DC. Working memory impairment as an endophenotypic marker of a schizophrenia diathesis. Schizophrenia Research: Cognition. 2014;1(3):127–36. 10.1016/j.scog.2014.09.005 25414816 PMC4234058

[14] Lee J, Park S. Working memory impairments in schizophrenia: a meta-analysis. Journal of abnormal psychology. 2005;114(4):599–611. 10.1037/0021-843X.114.4.599 . 16351383

[15] Forbes NF, Carrick LA, McIntosh AM, Lawrie SM. Working memory in schizophrenia: a meta-analysis. Psychological medicine. 2009;39(6):889–905. 10.1017/S0033291708004558 . 18945379

[16] Bora E, Murray RM. Meta-analysis of cognitive deficits in ultra-high risk to psychosis and first-episode psychosis: Do the cognitive deficits progress over, or after, the onset of psychosis? Schizophrenia bulletin. 2014;40(4):744–55. 10.1093/schbul/sbt085 23770934 PMC4059428

[17] Becker HE, Nieman DH, Wiltink S, Dingemans PM, van de Fliert JR, Velthorst E, et al Neurocognitive functioning before and after the first psychotic episode: does psychosis result in cognitive deterioration? Psychological medicine. 2010;40(10):1599–606. 10.1017/S0033291710000048 . 20132582

[18] Metzler S, Dvorsky D, Wyss C, Muller M, Gerstenberg M, Traber-Walker N, et al Changes in neurocognitive functioning during transition to manifest disease: comparison of individuals at risk for schizophrenic and bipolar affective psychoses. Psychological medicine. 2015:1–12. 10.1017/S0033291715000057 . 25640248

[19] Reichenberg A, Caspi A, Harrington H, Houts R, Keefe RS, Murray RM, et al Static and dynamic cognitive deficits in childhood preceding adult schizophrenia: a 30-year study. The American journal of psychiatry. 2010;167(2):160–9. 10.1176/appi.ajp.2009.09040574 20048021 PMC3552325

[20] Meier MH, Caspi A, Reichenberg A, Keefe RS, Fisher HL, Harrington H, et al Neuropsychological decline in schizophrenia from the premorbid to the postonset period: evidence from a population-representative longitudinal study. The American journal of psychiatry. 2014;171(1):91–101. 10.1176/appi.ajp.2013.12111438 24030246 PMC3947263

[21] Fatouros-Bergman H, Cervenka S, Flyckt L, Edman G, Farde L. Meta-analysis of cognitive performance in drug-naive patients with schizophrenia. Schizophrenia research. 2014;158(1–3):156–62. 10.1016/j.schres.2014.06.034 . 25086658

[22] Agnew-Blais J, Seidman LJ. Neurocognition in youth and young adults under age 30 at familial risk for schizophrenia: a quantitative and qualitative review. Cognitive neuropsychiatry. 2013;18(1–2):44–82. 10.1080/13546805.2012.676309 22998599 PMC3577989

[23] Snitz BE, Macdonald AW III, Carter CS. Cognitive deficits in unaffected first-degree relatives of schizophrenia patients: a meta-analytic review of putative endophenotypes. Schizophrenia bulletin. 2006;32(1):179–94. 10.1093/schbul/sbi048 16166612 PMC2632195

[24] Knowles EE, Mathias SR, McKay DR, Sprooten E, Blangero J, Almasy L, et al Genome-Wide Analyses of Working-Memory Ability: A Review. Current behavioral neuroscience reports. 2014;1(4):224–33. 10.1007/s40473-014-0028-8 25729637 PMC4339023

[25] Wardle MC, de Wit H, Penton-Voak I, Lewis G, Munafò MR. Lack of Association Between COMT and Working Memory in a Population-Based Cohort of Healthy Young Adults. Neuropsychopharmacology: official publication of the American College of Neuropsychopharmacology. 2013;38(7):1253–63. 10.1038/npp.2013.24 PMC3656369. 23337869 PMC3656369

[26] Fusar-Poli P, Deste G, Smieskova R, Barlati S, Yung AR, Howes O, et al Cognitive functioning in prodromal psychosis: a meta-analysis. Archives of general psychiatry. 2012;69(6):562–71. 10.1001/archgenpsychiatry.2011.1592 . 22664547

[27] De Herdt A, Wampers M, Vancampfort D, De Hert M, Vanhees L, Demunter H, et al Neurocognition in clinical high risk young adults who did or did not convert to a first schizophrenic psychosis: a meta-analysis. Schizophrenia research. 2013;149(1–3):48–55. 10.1016/j.schres.2013.06.017 . 23830855

[28] Broome MR, Day F, Valli I, Valmaggia L, Johns LC, Howes O, et al Delusional ideation, manic symptomatology and working memory in a cohort at clinical high-risk for psychosis: A longitudinal study. European Psychiatry. 2012;27(4):258–63. 10.1016/j.eurpsy.2010.07.008 20934858

[29] Choi JS, Park JY, Jung MH, Jang JH, Kang DH, Jung WH, et al Phase-specific brain change of spatial working memory processing in genetic and ultra-high risk groups of schizophrenia. Schizophrenia bulletin. 2012;38(6):1189–99. 10.1093/schbul/sbr038 21518920 PMC3494057

[30] Dickson H, Cullen AE, Reichenberg A, Hodgins S, Campbell DD, Morris RG, et al Cognitive impairment among children at-risk for schizophrenia. Journal of psychiatric research. 2014;50(1):92–9. 10.1016/j.jpsychires.2013.12.003 24373930

[31] Giakoumaki SG. Cognitive and prepulse inhibition deficits in psychometrically high schizotypal subjects in the general population: relevance to schizophrenia research. J Int Neuropsychol Soc. 2012;18(4):643–56. 10.1017/S135561771200029X . 22613272

[32] Brebion G, Bressan RA, Ohlsen RI, David AS. A model of memory impairment in schizophrenia: cognitive and clinical factors associated with memory efficiency and memory errors. Schizophrenia research. 2013;151(1–3):70–7. 10.1016/j.schres.2013.09.009 . 24113205

[33] Shakeel MK, Docherty NM. Neurocognitive predictors of source monitoring in schizophrenia. Psychiatry research. 2012;200(2–3):173–6. 10.1016/j.psychres.2012.06.014 22763089 PMC3500674

[34] Brebion G, Laroi F, Van der Linden M. Associations of hallucination proneness with free-recall intrusions and response bias in a nonclinical sample. Journal of clinical and experimental neuropsychology. 2010;32(8):847–54. 10.1080/13803391003596397 . 20373196

[35] Ochoa S, Haro JM, Huerta-Ramos E, Cuevas-Esteban J, Stephan-Otto C, Usall J, et al Relation between jumping to conclusions and cognitive functioning in people with schizophrenia in contrast with healthy participants. Schizophrenia research. 2014;159(1):211–7. 10.1016/j.schres.2014.07.026 . 25112159

[36] Garety P, Joyce E, Jolley S, Emsley R, Waller H, Kuipers E, et al Neuropsychological functioning and jumping to conclusions in delusions. Schizophrenia research. 2013;150(2–3):570–4. 10.1016/j.schres.2013.08.035 24075604 PMC3824078

[37] Freeman D, Startup H, Dunn G, Cernis E, Wingham G, Pugh K, et al Understanding jumping to conclusions in patients with persecutory delusions: working memory and intolerance of uncertainty. Psychological medicine. 2014;44(14):3017–24. 10.1017/S0033291714000592 . 25066636

[38] Leding JK. Working memory predicts the rejection of false memories. Memory. 2012;20(3):217–23. 10.1080/09658211.2011.653373 . 22292532

[39] Gerrie MP, Garry M. Individual differences in working memory capacity affect false memories for missing aspects of events. Memory. 2007;15(5):561–71. 10.1080/09658210701391634 . 17613798

[40] Radaelli D, Benedetti F, Cavallaro R, Colombo C, Smeraldi E. The reality monitoring deficit as a common neuropsychological correlate of schizophrenic and affective psychosis. Behavioral sciences. 2013;3(2):244–52. 10.3390/bs3020244 25379237 PMC4217621

[41] Korponay C, Nitzburg GC, Malhotra AK, DeRosse P. Positive and negative subclinical symptoms and MCCB performance in non-psychiatric controls. Schizophrenia Research: Cognition. 2014;1(4):175–9. 10.1016/j.scog.2014.09.002 25530948 PMC4266935

[42] Ziermans TB. Working memory capacity and psychotic-like experiences in a general population sample of adolescents and young adults. Frontiers in psychiatry. 2013;4:161 10.3389/fpsyt.2013.00161 24348432 PMC3847810

[43] Blanchard MM, Jacobson S, Clarke MC, Connor D, Kelleher I, Garavan H, et al Language, motor and speed of processing deficits in adolescents with subclinical psychotic symptoms. Schizophrenia research. 2010;123(1):71–6. 10.1016/j.schres.2010.05.028 20580205

[44] Kelleher I, Clarke MC, Rawdon C, Murphy J, Cannon M. Neurocognition in the extended psychosis phenotype: performance of a community sample of adolescents with psychotic symptoms on the MATRICS neurocognitive battery. Schizophrenia bulletin. 2013;39(5):1018–26. 10.1093/schbul/sbs086 22927672 PMC3756771

[45] Kelleher I, Murtagh A, Clarke MC, Murphy J, Rawdon C, Cannon M. Neurocognitive performance of a community-based sample of young people at putative ultra high risk for psychosis: support for the processing speed hypothesis. Cognitive neuropsychiatry. 2013;18(1–2):9–25. 10.1080/13546805.2012.682363 . 22991935

[46] Niarchou M, Zammit S, Walters J, Lewis G, Owen MJ, van den Bree MB. Defective processing speed and nonclinical psychotic experiences in children: longitudinal analyses in a large birth cohort. The American journal of psychiatry. 2013;170(5):550–7. 10.1176/appi.ajp.2012.12060792 23632836 PMC3662188

[47] Wickens TD. Elementary signal detection theory. Oxford: Oxford University Press; 2002.

[48] Bentall RP, Slade PD. Reality testing and auditory hallucinations: a signal detection analysis. The British journal of clinical psychology / the British Psychological Society. 1985;24 (Pt 3):159–69. . 4052663 10.1111/j.2044-8260.1985.tb01331.x

[49] Boyd A, Golding J, Macleod J, Lawlor DA, Fraser A, Henderson J, et al Cohort Profile: the 'children of the 90s'—the index offspring of the Avon Longitudinal Study of Parents and Children. International journal of epidemiology. 2013;42(1):111–27. 10.1093/ije/dys064 22507743 PMC3600618

[50] Fraser A, Macdonald-Wallis C, Tilling K, Boyd A, Golding J, Davey Smith G, et al Cohort Profile: the Avon Longitudinal Study of Parents and Children: ALSPAC mothers cohort. International journal of epidemiology. 2013;42(1):97–110. 10.1093/ije/dys066 22507742 PMC3600619

[51] Schedules for clinical assessment in neuropsychiatry. 2. ed Geneva: World Health Organization, Division of Mental Health; 1994.

[52] Macmillan NA, Creelman CD. Detection theory: a user's guide. 2nd ed ed. Mahwah N.J.; London: Lawrence Erlbaum Associates; 2005.

[53] Stanislaw H, Todorov N. Calculation of signal detection theory measures. Behavior research methods, instruments, & computers: a journal of the Psychonomic Society, Inc. 1999;31(1):137–49. . 10495845 10.3758/bf03207704

[54] Kumar CT, Christodoulou T, Vyas NS, Kyriakopoulos M, Corrigall R, Reichenberg A, et al Deficits in visual sustained attention differentiate genetic liability and disease expression for schizophrenia from Bipolar Disorder. Schizophrenia research. 2010;124(1–3):152–60. 10.1016/j.schres.2010.07.006 . 20674278

[55] Dawkins L, Powell JH, West R, Powell J, Pickering A. A double-blind placebo-controlled experimental study of nicotine: II—Effects on response inhibition and executive functioning. Psychopharmacology. 2007;190(4):457–67. 10.1007/s00213-006-0634-6 . 17205318

[56] Lewis G. Assessing psychiatric disorder with a human interviewer or a computer. Journal of epidemiology and community health. 1994;48(2):207–10. 8189180 10.1136/jech.48.2.207 PMC1059935

[57] Lewis G, Pelosi AJ, Araya R, Dunn G. Measuring psychiatric disorder in the community: a standardized assessment for use by lay interviewers. Psychological medicine. 1992;22(2):465–86. . 1615114 10.1017/s0033291700030415

[58] Wechsler D, Golombok J, Rust J. WISC-IIIUK: Wechsler Intelligence Scale for Children (Sidcup, UK: The Psychological Corporation). 1992.

[59] Bowen E, Heron J, Waylen A, Wolke D, Team AS. Domestic violence risk during and after pregnancy: findings from a British longitudinal study. BJOG: an international journal of obstetrics and gynaecology. 2005;112(8):1083–9. 10.1111/j.1471-0528.2005.00653.x . 16045522

[60] Legleye S, Piontek D, Kraus L, Morand E, Falissard B. A validation of the Cannabis Abuse Screening Test (CAST) using a latent class analysis of the DSM-IV among adolescents. International journal of methods in psychiatric research. 2013;22(1):16–26. 10.1002/mpr.1378 . 23519957 PMC6878590

[61] Legleye S, Karila L, Beck F, Reynaud M. Validation of the CAST, a general population Cannabis Abuse Screening Test. Journal of Substance Use. 2007;12(4):233–42. 10.1080/14659890701476532

[62] Royston P. Multiple imputation of missing values: further update of ice, with an emphasis on interval censoring. Stata Journal. 2007;7(4):445–64.

[63] Ziermans TB. Working memory capacity and psychotic-like experiences in a general population sample of adolescents and young adults. Frontiers in Psychiatry. 2013;4(DEC). 10.3389/fpsyt.2013.00161 PMC3847810 24348432

[64] Nuechterlein KH, Green MF, Kern RS, Baade LE, Barch DM, Cohen JD, et al The MATRICS Consensus Cognitive Battery, part 1: test selection, reliability, and validity. The American journal of psychiatry. 2008;165(2):203–13. 10.1176/appi.ajp.2007.07010042 . 18172019

[65] Perlstein WM, Dixit NK, Carter CS, Noll DC, Cohen JD. Prefrontal cortex dysfunction mediates deficits in working memory and prepotent responding in schizophrenia. Biological psychiatry. 2003;53(1):25–38. . 12513942 10.1016/s0006-3223(02)01675-x

[66] Callicott JH, Bertolino A, Mattay VS, Langheim FJ, Duyn J, Coppola R, et al Physiological dysfunction of the dorsolateral prefrontal cortex in schizophrenia revisited. Cerebral cortex. 2000;10(11):1078–92. . 11053229 10.1093/cercor/10.11.1078

[67] Carter CS, Perlstein W, Ganguli R, Brar J, Mintun M, Cohen JD. Functional hypofrontality and working memory dysfunction in schizophrenia. The American journal of psychiatry. 1998;155(9):1285–7. . 9734557 10.1176/ajp.155.9.1285

[68] Schmidt-Hansen M, Honey RC. Working memory and multidimensional schizotypy: dissociable influences of the different dimensions. Cognitive neuropsychology. 2009;26(7):655–70. 10.1080/02643291003644501 . 21793793

[69] Koychev I, McMullen K, Lees J, Dadhiwala R, Grayson L, Perry C, et al A validation of cognitive biomarkers for the early identification of cognitive enhancing agents in schizotypy: a three-center double-blind placebo-controlled study. Eur Neuropsychopharmacol. 2012;22(7):469–81. 10.1016/j.euroneuro.2011.10.005 . 22137565

[70] Nuechterlein KH, Green MF, Calkins ME, Greenwood TA, Gur RE, Gur RC, et al Attention/vigilance in schizophrenia: performance results from a large multi-site study of the Consortium on the Genetics of Schizophrenia (COGS). Schizophrenia research. 2015;163(1–3):38–46. 10.1016/j.schres.2015.01.017 25749017 PMC4382444

[71] Brebion G, David AS, Ohlsen R, Jones HM, Pilowsky LS. Visual memory errors in schizophrenic patients with auditory and visual hallucinations. J Int Neuropsychol Soc. 2007;13(5):832–8. 10.1017/S135561770707107X . 17697414

[72] Rankin PM, O'Carroll PJ. Reality discrimination, reality monitoring and disposition towards hallucination. The British journal of clinical psychology / the British Psychological Society. 1995;34 (Pt 4):517–28. . 8563659 10.1111/j.2044-8260.1995.tb01486.x

[73] Brebion G, David AS, Jones H, Pilowsky LS. Hallucinations, negative symptoms, and response bias in a verbal recognition task in schizophrenia. Neuropsychology. 2005;19(5):612–7. 10.1037/0894-4105.19.5.612 . 16187879

[74] Keilp JG, Sackeim HA, Mann JJ. Correlates of trait impulsiveness in performance measures and neuropsychological tests. Psychiatry research. 2005;135(3):191–201. 10.1016/j.psychres.2005.03.006 . 15996748

[75] Waters FA, Badcock JC, Maybery MT, Michie PT. Inhibition in schizophrenia: association with auditory hallucinations. Schizophrenia research. 2003;62(3):275–80. . 12837525 10.1016/s0920-9964(02)00358-4

[76] Dolgov I, McBeath MK. A signal-detection-theory representation of normal and hallucinatory perception. Behavioral and Brain Sciences. 2005;28(06):761–2. 10.1017/S0140525X05260132

[77] Vercammen A, de Haan EH, Aleman A. Hearing a voice in the noise: auditory hallucinations and speech perception. Psychological medicine. 2008;38(8):1177–84. 10.1017/S0033291707002437 . 18076771

[78] Waters F, Allen P, Aleman A, Fernyhough C, Woodward TS, Badcock JC, et al Auditory hallucinations in schizophrenia and nonschizophrenia populations: a review and integrated model of cognitive mechanisms. Schizophrenia bulletin. 2012;38(4):683–93. 10.1093/schbul/sbs045 22446568 PMC3406530

[79] Garwood L, Dodgson G, Bruce V, McCarthy-Jones S. A preliminary investigation into the existence of a hypervigilance subtype of auditory hallucination in people with psychosis. Behavioural and cognitive psychotherapy. 2015;43(1):52–62. 10.1017/S1352465813000714 . 23962410

[80] Dodgson G, Gordon S. Avoiding false negatives: are some auditory hallucinations an evolved design flaw? Behavioural and cognitive psychotherapy. 2009;37(3):325–34. 10.1017/S1352465809005244 . 19371459

[81] Holmes AJ, MacDonald A III, Carter CS, Barch DM, Andrew Stenger V, Cohen JD. Prefrontal functioning during context processing in schizophrenia and major depression: an event-related fMRI study. Schizophrenia research. 2005;76(2–3):199–206. 10.1016/j.schres.2005.01.021 . 15949653

[82] MacDonald AW III, Carter CS, Kerns JG, Ursu S, Barch DM, Holmes AJ, et al Specificity of prefrontal dysfunction and context processing deficits to schizophrenia in never-medicated patients with first-episode psychosis. The American journal of psychiatry. 2005;162(3):475–84. 10.1176/appi.ajp.162.3.475 . 15741464

[83] Barch DM, Carter CS, MacDonald AW III, Braver TS, Cohen JD. Context-processing deficits in schizophrenia: diagnostic specificity, 4-week course, and relationships to clinical symptoms. Journal of abnormal psychology. 2003;112(1):132–43. . 12653421

[84] Garety P, Freeman D, Jolley S, Ross K, Waller H, Dunn G. Jumping to conclusions: The psychology of delusional reasoning. Advances in Psychiatric Treatment. 2011;17(5):332–9. 10.1192/apt.bp.109.007104

[85] Broome MR, Johns LC, Valli I, Woolley JB, Tabraham P, Brett C, et al Delusion formation and reasoning biases in those at clinical high risk for psychosis. British Journal of Psychiatry. 2007;191(SUPPL. 51):s38–s42. 10.1192/bjp.191.51.s38 18055936

[86] Moritz S, Woodward TS. Jumping to conclusions in delusional and non-delusional schizophrenic patients. The British journal of clinical psychology / the British Psychological Society. 2005;44(Pt 2):193–207. 10.1348/014466505X35678 . 16004654

[87] Winton-Brown TT, Broome MR, Allen P, Valli I, Howes O, Garety PA, et al Misattributing speech and jumping to conclusions: a longitudinal study in people at high risk of psychosis. European psychiatry: the journal of the Association of European Psychiatrists. 2015;30(1):32–7. 10.1016/j.eurpsy.2014.09.416 . 25511317

[88] Ross RM, McKay R, Coltheart M, Langdon R. Jumping to Conclusions About the Beads Task? A Meta-analysis of Delusional Ideation and Data-Gathering. Schizophrenia bulletin. 2015 10.1093/schbul/sbu187 . 25616503 PMC4535629

[89] van der Leer L, Hartig B, Goldmanis M, McKay R. Delusion proneness and 'jumping to conclusions': relative and absolute effects. Psychological medicine. 2015;45(6):1253–62. 10.1017/S0033291714002359 . 25272196

[90] Fletcher PC, Frith CD. Perceiving is believing: a Bayesian approach to explaining the positive symptoms of schizophrenia. Nature reviews Neuroscience. 2009;10(1):48–58. 10.1038/nrn2536 . 19050712

[91] Adams RA, Stephan KE, Brown HR, Frith CD, Friston KJ. The computational anatomy of psychosis. Frontiers in psychiatry. 2013;4:47 10.3389/fpsyt.2013.00047 23750138 PMC3667557

[92] Anticevic A, Murray JD, Barch DM. Bridging Levels of Understanding in Schizophrenia Through Computational Modeling. Clinical psychological science: a journal of the Association for Psychological Science. 2015;3(3):433–59. 10.1177/2167702614562041 25960938 PMC4421907

[93] Avery MC, Krichmar JL. Improper activation of D1 and D2 receptors leads to excess noise in prefrontal cortex. Frontiers in computational neuroscience. 2015;9:31 10.3389/fncom.2015.00031 25814948 PMC4356073

[94] Okimura T, Tanaka S, Maeda T, Kato M, Mimura M. Simulation of the capacity and precision of working memory in the hypodopaminergic state: Relevance to schizophrenia. Neuroscience. 2015;295:80–9. 10.1016/j.neuroscience.2015.03.039 . 25818554

[95] Braver TS, Barch DM, Cohen JD. Cognition and control in schizophrenia: a computational model of dopamine and prefrontal function. Biological psychiatry. 1999;46(3):312–28. . 10435197 10.1016/s0006-3223(99)00116-x

[96] Fleming SM, Dolan RJ, Frith CD. Metacognition: computation, biology and function. Philosophical transactions of the Royal Society of London Series B, Biological sciences. 2012;367(1594):1280–6. 10.1098/rstb.2012.0021 22492746 PMC3318771

[97] Moutoussis M, Bentall RP, El-Deredy W, Dayan P. Bayesian modelling of Jumping-to-Conclusions bias in delusional patients. Cognitive neuropsychiatry. 2011;16(5):422–47. 10.1080/13546805.2010.548678 . 21480015

[98] Locke HS, Braver TS. Motivational influences on cognitive control: behavior, brain activation, and individual differences. Cognitive, affective & behavioral neuroscience. 2008;8(1):99–112. . 18405050 10.3758/cabn.8.1.99

[99] Bang M, Kim KR, Song YY, Baek S, Lee E, An SK. Neurocognitive impairments in individuals at ultra-high risk for psychosis: Who will really convert? The Australian and New Zealand journal of psychiatry. 2015;49(5):462–70. 10.1177/0004867414561527 . 25425742

